# Pristine biochar performance investigation to remove metals in primary and secondary treated municipal wastewater for groundwater recharge application

**DOI:** 10.1371/journal.pone.0278315

**Published:** 2022-12-06

**Authors:** Yohanna Haile Fseha, Banu Sizirici, Ibrahim Yildiz, Cafer Yavuz

**Affiliations:** 1 Department of Civil Infrastructure and Environmental Engineering, Khalifa University, Abu Dhabi, United Arab Emirates; 2 Department of Chemistry, Khalifa University, Abu Dhabi, United Arab Emirates; 3 Advanced Membranes and Porous Materials Center, King Abdullah University of Science and Technology, Thuwal, Kingdom of Saudi Arabia; King Saud University, SAUDI ARABIA

## Abstract

In this study, pristine biochar derived from date palm at 500°C was used in batch reactors (simulating blending adsorbent in aeration tank) and fixed-bed columns (simulating holding adsorbent in fixed-bed reactors). The removal performance of the biochar was assessed toward single and mixed-metal solutions as well as synthetic primary and secondary treated wastewater for copper (Cu^2+^), iron (Fe^2+^), nickel (Ni^2+^) and zinc (Zn^2+^). The order of maximum adsorption capacities of the metal ions at pH 7 followed: Fe^2+^ (2.92/2.94 mg/g)>Cu^2+^(2.69/2.78 mg/g) >Zn^2+^(2.03/2.19 mg/g)>Ni^2+^(1.69/1.02 mg/g) in single/mixed-metal solutions and Zn^2+^(2.91/11.26 mg/g)>Fe^2+^(0.60/5.29 mg/g)>Cu^2+^(0.56/5.05 mg/g)>Ni^2+^(0.13/2.02 mg/g) in synthetic primary/secondary treated wastewater. Blending biochar in aeration tank reduced metal concentrations. The metal ion concentrations in the final effluent were below the World Health Organization drinking water limits (2, 0.3, 0.1 and 3 mg/L for Cu^2+^, Fe^2+^, Ni^2+^ and Zn^2+^, respectively) suggesting that treated secondary wastewater can be spread into potable aquifers following disinfection. The Freundlich and the Pseudo-second order models fit best the batch experimental data. Experimental data from column analysis fit well to the Thomas model. The adsorption of metal ions on the surface of biochar was confirmed by Scanning electron microscopy, Energy dispersive X-ray studies, X-ray photoelectron spectroscopy, Fourier transform infrared spectroscopy and X-ray diffraction. Desorption studies using different eluents demonstrated the reusability potential of the studied biochar.

## Introduction

Water reuse becomes an essential requirement to meet the current water demand without compromising the needs of the future generations as water resources become scarce [1]. Reclaimed water can be used for irrigation of crops and urban areas, creation of wetlands, cooling water for industries and groundwater recharge with the aid of injection wells or infiltration basins [2]. Groundwater recharge replenishes groundwater level, protects aquifers against saltwater intrusion, and stores water for future use [3]. Treatment requirements for groundwater recharge vary depending on the final usage of groundwater (non-potable/ indirect potable use) [4]. Groundwater recharge by spreading into potable aquifers for indirect potable reuse requires secondary treatment of municipal wastewater with disinfection and filtration together with probable advanced treatment [2]. Moreover, the depth to groundwater should be at least 2 m; reclaimed water should be retained at least 6 months prior to withdrawal; and reclaimed water quality should meet drinking water standards after percolation. The composition of secondary treated wastewater varies based on the influent concentrations. Due to illegally dumped industrial wastewaters, the concentrations of heavy metal ions increase in municipal wastewater [5]. According to some studies Cu^2+^, Fe^2+^, Ni^2+^ and Zn^2+^ from electroplating and metal finishing industries are the most frequently observed metal ions in secondary treated wastewater concentrations ranging from <0.3 to 4.2 mg/L [6–8]. Hence, in order to protect human health and also to preserve the integrity of natural water reservoirs, wastewater effluents containing such metals need to be treated before groundwater recharge so as they meet WHO drinking water limits of 2, 0.3, 0.1 and 3 mg/L respectively for Cu^2+^, Fe^2+^, Ni^2+^ and Zn^2+^ [9, 10].

Most municipal wastewater treatment plants use activated sludge systems which are not capable of removing metal ions from influent effectively [5]. Therefore, groundwater recharge by surface spreading operations requires adsorption, tertiary granular-medium filtration to remove inorganics and organics furtherly, and disinfection of municipal wastewater [11]. Adsorption has gained popularity as an efficient, versatile and cost-effective technique with minimal sludge production for the effective decontamination of water and wastewater [12]. The most common adsorbent used in adsorption systems is activated carbon, however, it has high production and regeneration costs which necessitates the quest for more economical and environmentally friendly adsorbents [12]. As such, alternative adsorbents of biological, industrial and agricultural origins have been investigated because they are widely available, low in cost and have potential in the adsorption of contaminants owing to their physiochemical characteristics [13]. Recently, studies have investigated biochar, which is a carbonaceous, porous product obtained from the pyrolysis of locally available materials/waste in limited or no oxygen environment, as a cost-effective and eco-friendly alternative adsorbent [14, 15]. Biochar is found to be advantageous in adsorbing and removing pollutants including heavy metal ions due to its favourable characteristics such as high surface area, chemical stability, and abundance of functional groups [16]. Biochar derived from plant waste, particularly, is highly advantageous in the removal of heavy metals from aqueous solutions due to its higher porosity and larger internal surface area [17]. Heavy metal ion removal by biochar via batch studies has been the topic of investigation of various studies. A study showed that, adsorption capacities of corn straw biochar were 12.5.0 and 11.0 mg/g for Cu^2+^ and Zn^2+^ and hardwood biochar were 6.8 and 4.5 mg/g for Cu^2+^ and Zn^2+^ [18]. The adsorption capacities of bone char pyrolyzed at 500°C were 47.6 and 34.7 mg/g for Cu^2+^ and Zn^2+^ [19]. Mohan et al. [20] demonstrated that the adsorption capacities of oak wood, pine bark, and oak bark for Cd^2+^ were 0.4, 0.5 and 5.4 mg/g, respectively. Almond shell biochar showed adsorption capacities of 22.2 mg/g for Ni^2+^ and 28.09 mg/g for Co^2+^ [21]. Otero et al. [22] reported that sewage sludge’s adsorption capacities for Cu^2+^, Cr^2+^ and Pb^2+^ were 6.7, 3, and 40.3 mg/g, respectively. According to Pellera et al. [23], adsorption capacities of rice husk, olive pomace, orange waste and compost pyrolyzed at 600°C for Cu^2+^ were 0.3, 0.7, 0.4 and 3.4 mg/g, respectively. Although batch adsorption studies provide useful information on adsorption parameters, both batch and column studies are necessary to determine the optimum conditions of the adsorbent. However, studies using biochar in continuous-mode column operations for the removal of multiple heavy metals in single and quaternary systems are limited. Such investigations are necessary because heavy metals are not found alone in the wastewater and often interact with one another and other matrix components; hence, it is important to study their adsorption characteristics in single as well as multi-component systems [24]. Biochar obtained from date seed showed an adsorption capacity of 1.45 mg/g for Pb^2+^ in a column study [25]. In another column study, the adsorption capacities of biochar derived from spent coffee grounds were 31.15 mg/g of Cu^2+^ and 213.23 mg/g of Pb^2+^ [26]. Maximum adsorption capacities of *Tectona grandis* leaves-derived biochar in column were found 26.99 mg/g of Ni^2+^ and 23.63 mg/g of Co^2+^ [27]. Abdallah et al. [28] reported that the adsorption capacities of spent mushroom compost biochar for the removal of Zn^2+^, Cu^2+^ and Pb^2+^ were 0.6, 1.11 and 12.7 mg/g, respectively. Yet, these studies are not very representative of adsorbent performance in municipal wastewater treatment.

Treatment of municipal wastewater with adsorbent involves 1) adding the adsorbent material directly to the aeration tank 2) adding it into the fixed-bed reactor [2]. To the best of our knowledge there is no other study that uses biochar derived from date palm waste, specifically the fronds and leaves, for the adsorptive removal of multiple metals from single, mixed-metal solutions, and synthetic primary and secondary treated municipal wastewater (in presence of other inorganics and organics) using both batch and fixed-bed reactor/column studies for groundwater recharge applications. Therefore, in this study pristine date palm biochar pyrolyzed at 500°C was utilized in batch reactor to determine kinetics and the optimum conditions to remove metal ions (Cu^2+^, Fe^2+^, Ni^2+^ Zn^2+^) in single/mixed-metal solutions. The optimum conditions determined from the kinetics study were applied to simulated aeration tank systems to remove metal ions from primary treated wastewater. The adsorption dynamics were assessed in fixed-bed column studies using single and mixed-metal solutions. The optimum conditions obtained from column studies were applied to remove metal ions from secondary treated wastewater. Desorption studies were performed to assess the regeneration of the pristine date palm biochar as a potential commercial adsorbent. Based on the results of this study, date palm waste-derived biochar can be utilized in wastewater treatment plants as a low-cost adsorbent to remove metal ions efficiently.

## Materials and methods

### Methods of preparation of pristine biochar and metal /synthetic wastewater solutions

Previously, we reported that the biochars derived from date palm frond and date palm leaf at 500°C (named as Frond 500 and Leaf 500 respectively) exhibited higher removal rates of metal ions at pH > 6 [17]. Therefore, a mixture of Leaf 500 and Frond 500 biochars (named as LF500) as 50/50% by weight was used as the adsorbent in this study. The preparation of biochar was adopted from our previous study [17]. Briefly, the fronds and leaves of date palm waste were collected from the backyard of Khalifa University, Abu Dhabi, United Arab Emirates; shortly after collection they were sun-dried, chopped and prepared for pyrolysis in a muffle furnace (Vulcan 3–550, Neytech, Dentsply, USA) at a temperature of 500°C and 8°C/min temperature increment. After completion of pyrolysis, the biochar samples were left to cool inside the furnace. Later, the frond and leaf biochar samples were ground and sieved to obtain a particle size of 0.15 mm for batch studies and 1 mm for column studies. The sieved biochar samples were washed with deionized water (Milli-Q, Progard TS2 5 UV) and dried at 105°C for two hours before being placed inside air-tight containers in desiccators.

Stock solutions of 5 mg/L of Cu^2+^, Fe^2+^, Ni^2+^ and Zn^2+^ were prepared using CuSO_4_.5H_2_O, FeSO_4_.7H_2_O, NiCl_2_.6H_2_O and ZnCl₂ for the batch and column studies. All chemicals were of analytical grade and purchased from Merck, Darmstadt, Germany. This particular concentration was used in this study because concentrations of the studied metals in secondary treated wastewater range from <0.3 to 4.2 mg/L as aforementioned [6–8]. The synthetic wastewater was prepared according to reported protocol [29]. The wastewater was diluted 40 times; it was spiked with metal salts; and chemical concentrations were found 503± 3 mg/L for COD, 191 ±1 mg/L for BOD, 3 mg/L for Cu^2+^, 1 mg/L for Fe^2+^, 4 mg/L for Zn^2+^ and 0.2 mg/L for Ni^2+^ as pretreated wastewater. The chemical composition of 100 times diluted wastewater sample were 109± 4 mg/L of COD, 27.25 ± 1 mg/L of BOD, 3 mg/L of Cu^2+^, 1 mg/L of Fe^2+^, 4 mg/L of Zn^2+^ and 0.2 mg/L of Ni^2+^ as secondary treated wastewater.

### Characterization of adsorbent

The LF500 biochar samples were characterized before and after adsorption of metal ions using scanning electron microscopy (SEM) (JEOL JSM-7610F FEG-SEM, Tokyo, Japan) coupled with energy dispersive X-ray (EDX), Fourier transform infrared spectrometry (FT-IR) (Perkin Elmer Spectrum Two, Waltham, MA, USA), and X-Ray diffraction (XRD) (Bruker D2 Phaser, Billerica, MA, USA)). The X-ray photoelectron spectroscopy (XPS) measurements were carried out in a Kratos Axis Supra DLD spectrometer, UK (hv = 1,486.6 eV) operating at 75 W, a multichannel plate, and delay line detector under a vacuum of 1 × 10−8 mbar equipped with a monochromatic Al K X-ray source. Survey and high-resolution spectra were performed at fixed analyzer pass energies of 160 and 20 eV, respectively. The elemental analysis and surface area analysis via Brunauer–Emmett–Teller method of date palm LF500 biochar samples were explained in detail in our previous study [17].

For the quantitative determination of the functional groups present on the surface of LF500 biochar, Boehm’s titration was conducted according to methods proposed in the literature [30, 31]. Briefly, 0.25 g of LF500 biochar samples was added individually to 12.5 mL of the following solutions: 0.05 M of HCl, NaOH, NaHCO_3_ and Na_2_CO_3_. The samples were left to shake for 48 h at 50 rpm and room temperature after which they were filtered using Whatman 0.45-micron filter paper. 10 mL of each of the filtrates was transferred to new tubes containing various amounts of 0.05 M HCl while no solution was added to the filtrate containing HCl. After, a few drops of methyl red indicator was added to the samples followed by titration with 0.05 M NaOH until the endpoint (yellow) was reached. To ensure accuracy, the experiment was performed in duplicate. Using mathematical relationships, the number of functional groups on the surface of LF500 biochar (n) in mmol/g was computed [30]. As NaHCO_3_ reacts only with carboxylic groups, n (NaHCO_3_) can directly give the quantity of carboxyl groups present [31]. As Na_2_CO_3_ reacts with both the carboxyl and lactonic groups, the difference between n (Na_2_CO_3_) and n (NaHCO_3_) gives the lactonic groups present [32]. As for NaOH, it reacts with carboxyl, lactonic and phenolic groups and hence, the difference n (NaOH) and n (Na_2_CO_3_) yields the phenolic groups present [30].

### Batch study set up

The influence of pH, contact time, and dosage were studied on the removal and adsorption capacities of biochar for Cu^2+^, Fe^2+^, Ni^2+^ and Zn^2+^ in single, mixed-metal solutions and synthetic wastewater. 0.1 g of LF500 biochar (0.15 mm of size) was mixed with 50 mL of 5 mg/L of single and mixed-metal solutions in tightly closed Erlenmeyer flasks and shaken at room temperature for 20 h at 200 rpm on a shaker (Orbi-Shaker™ Orbital Shaker, BenchMark Scientific, USA) in duplicates. The pH was adjusted using 0.1 M of NaOH or HCl. The influence of pH on adsorption was analyzed by varying pH to 2, 4, 6, 7, 8 and 10. The influence of contact time was analyzed for 1, 5, 15, 30, 60, 120, 240, 480, 960, 1200, 1440, and 2160 min at 200 rpm at room temperature while keeping the other parameters fixed (dosage = 0.1 g and pH 7). The effect of dosage was investigated by changing the mass of biochar (0.05, 0.1, 0.2 and 0.4 g) while at the same time keeping the other parameters fixed (pH 7, contact time = 20 h). Optimum conditions obtained from single and mixed-metal solutions were used for the simulation of blending biochar in aeration tank in activated sludge system for pretreated wastewater. 0.1 g of LF500 biochar was added to 50 mL synthetic wastewater at pH 7, and the resulting mixture was shaken for 20 h. Samples were filtered using Whatman 0.45-micron filter paper, and the concentrations of the metal ions in influent and effluent were analyzed using ultraviolet–visible (UV vis) spectrometry (Hach, Lange DR6000, Dusseldorf, Germany) and inductively coupled plasma mass spectrometry (ICP-MS), (PerkinElmer NexION 350 X dual channel, Wellesley, MA, USA).

The removal efficiencies of the metals was obtained using Eq (1) [33]:

$$\%\;removal=\frac{C_{0}-C_{e}}{C_{0}}∙100$$ (1)

where, C_0_ = metal concentration of the influent (mg/L), C_e_ = metal concentration of the effluent (mg/L)

The pristine biochar’s metal sorption capacity, q_e_ (mg/g), was calculated as shown by Eq (2) [17]:

$$q_{e}=(C_{0}-C_{e})∙\frac{V}{m}$$ (2)

where, V is the sample’s volume (L) and m represents the weight of the LF500 biochar (g).

#### Adsorption isotherms and kinetics

The linearized and non-linear Langmuir, Freundlich, and Temkin models were utilized to fit the experimental data, as shown by Eqs (3)–(8) respectively. It has been stated that linearizing non-linear expressions to linear form is prone to error as the transformed equations may not satisfy the assumptions of least square analysis [34].


$$\frac{1}{q_{e}}=\frac{1}{q_{m}}+\frac{1}{q_{m}*K_{L}}∙\frac{1}{C_{e}}$$ (3)

$$q_{e}=\left(q_{m}∙K_{L}∙C_{e}\right)/\left(1+K_{L}∙C_{e}\right)$$ (4)

Where, q_m_ is the maximum adsorption capacity of the biochar (mg/g); K_L_ is adsorption affinity constant, (L/mg); q_e_ is the equilibrium concentration of the metal present in solution (mg/g).


$$\log\;q_{e}=\log\;K_{f}+\frac{1}{n}\log\;C_{e}$$ (5)

$$q_{e}=K_{f}∙C_{e}^{1/n}$$ (6)

Where, n is the adsorption intensity (dimensionless), K_F_ = Freundlich constant (mg/g).

The adsorption intensity (n) ranging between 0 and 1, is an indication of a heterogeneous surface and higher adsorption capacity as it approaches to zero. Values that are in the range of 1<n<10 are an indication of favourable adsorption [35].


$$q_{e}=B\;\ln\;A+B\;\ln\;C_{e}$$ (7)

$$q_{e}=B∙\ln{(A∙C}_{e})$$ (8)

Where, A and B are Temkin constants related to the equilibrium binding energy (L/mg) and the heat of adsorption (J/mol), respectively.

The pseudo-first, -second order, and Elovich models describe the adsorption kinetics as shown in Eqs (9)–(14), respectively:

$$\log{(q}_{e}-q_{t})=log\;q_{e}-{((k}_{1}/2.303)∙t)$$ (9)

$$q_{t}=q_{e}(1-e^{-k_{1}∙t})$$ (10)

Where, k_1_ is the rate constant of first-order adsorption (1/min), q_t_ denotes the quantity of metal ion adsorbed (mg/g) at a specific time, t represents the time (min).


$$\frac{t}{q_{t}}=\frac{1}{h}+\frac{1}{q_{e}}∙t$$ (11)

$$q=(K_{2}∙q_{e}^{2}∙t)/(1+K_{2}∙q_{e}∙t)$$ (12)

Where, K_2_ is the overall rate constant for the pseudo-second order model (g/mg/min), and h is the initial rate constant (mg/g/min).


$$q_{t}=\frac{1}{\beta }ln\;(\alpha \;\beta )+\frac{1}{\beta }ln(t)$$ (13)

q=1/βln(1+αβt) (14)

Where, α is the initial adsorption rate for Elovich model (mg/g/min) and β is the desorption constant (g/mg).

The correlation coefficient (R^2^) analyzes the linear relationship between the experimental data and the isotherm/kinetic. However, R^2^ alone cannot be used as a reliable statistical measure validating the isotherm/kinetics data because it can yield high values, therefore Chi-square (χ^2^) and root mean square error (RMSE) were used as statistical measures as well [36]. The lowest χ^2^ and RMSE indicate the highest correlations.

### Fixed-bed column setup

Chromatography columns having 1.5 cm inner diameter and 8 cm height were used as fixed-bed columns in duplicate. The adsorption dynamics of LF500 biochar (1 mm of size) was studied by varying (I) the initial influent metal concentrations from 2.5 to 5 mg/L in single and mixed -metal solutions, keeping bed height at 1 cm and flow rate at 10 mL/min (II) the bed depth of the biochar from 0.5 to 1 cm, keeping influent concentration at 5 mg/L and flow rate at 10 mL/min and (III) the flow rates from 10 to 20 mL/min, keeping bed height at 1 cm and influent concentration at 5 mg/L. The optimum conditions (bed depth of 1 cm and flow rate of 10 mL/min) observed from the single and mixed-metals column studies were applied to the fixed-bed columns to treat secondary treated wastewater. A layer of gravel (2 mm size) having 1 cm thickness was placed at the bottom of the columns in order to block the leakage of biochar. A multichannel peristaltic pump (Cole Palmer MasterFlex L/S) was used to feed single, mixed-metal solution, and synthetic wastewater which were constantly stirred using a magnetic stirrer. The pH of the metal solutions was kept at 7.0. Effluents were collected at different times, and metal ion concentrations were determined by ICP-MS and UV vis spectrometers. Once the metal concentrations in the effluent reached to 95% of the influent concentrations, the column study was stopped.

Removal rates of metals by biochar in the columns were calculated as shown in Eq (15):

$$\%\;Removal=\frac{q_{total}}{m_{total}}*100$$ (15)

Whereby, q_total_ denotes the biochar’s adsorption capacity (mg/g) and m_total_ indicates the amount of metal ion in total that goes into the column.

q_total_ and m_total_ can be calculated as follows:

$$q_{total}=\frac{QA}{1000}=\frac{Q}{1000}=\int_{t=0}^{t=t\;total}C_{ad}\;dt$$ (16)

$$m_{total}=\frac{C_{0}*Q*t_{total}}{1000}$$ (17)

where, C_ad_ denotes the adsorbed metal concentration (mg/L) which is the difference between the influent and effluent metal concentrations; Q is the flow rate (L/min); t_total_ is the detention time in the column in min and A denotes the area that is under the breakthrough curve.

In order to calculate the experimental equilibrium uptake, q_eq(exp)_, Eq (18) was used:

$$q_{eq(exp)}=\frac{q_{total}}{m}$$ (18)

Where, m denotes the mass of the biochar in the column (g).

The linearized Thomas model was utilized to analyze the adsorption dynamics of the metal ions in the column which is given by Eq (19).

$$ln(\frac{C_{0}}{C_{t}}-1)=k_{th}*q_{0}*\frac{m}{Q}-k_{th}*C_{0}*t$$ (19)

where, q_0_ is the metal uptake at equilibrium per gram of biochar (mg/g) and k_th_ is the Thomas rate constant (L/mg/h).

### Regeneration of biochar

LF500 biochar was regenerated by two consecutive adsorption and desorption cycles. 0.1 g of pristine biochar was added to 50 mL of single and mixed-metal solutions and mixed at 200 rpm at pH 7 for 20 h. The filtrate was analyzed to calculate the amount of metal ions that have adsorbed on the biochar. Then, the biochar sample was added to 50 mL of 0.1 M HCl solution and mixed at 200 rpm for 20 h. Afterward, the solution was filtered to separate the pristine biochar from the solution. The filtrate was analyzed to determine the amount of metal ions desorbed from pristine biochar. The remaining biochar was dried to be reused in the next adsorption-desorption cycle. This procedure was repeated with two other eluents- 0.1 M HNO_3_ and 0.1 M NaOH to compare the effect of different eluents on the reusability of LF500 biochar.

## Results

### Batch study

Lower adsorption capacities and removal efficiencies were observed for solutions having pH<6 for all single and mixed-metal solutions as shown in S1 Table. It was shown that the point of zero charge (pH_PZC_) was pH 7 for LF500 biochar suggesting that below pH 7 the biochar gains positive charges due to the protonation of acidic functional groups causing electrostatic repulsion of metal cations [37]. Furthermore, at lower pH values, metal ions are in competition with abundant H^+^ ions for the available binding sites [38]. The highest average adsorption capacities and removal efficiencies were observed at pH 7 with the following order: Fe^2+^ (2.92 mg/g, 99.15%) >Cu^2+^ (2.69 mg/g, 94.88%) >Zn^2+^ (2.03 mg/g, 93.49%) > Ni^2+^ (1.69 mg/g, 75.46%) in single-metal solutions, and Fe^2+^ (2.94 mg/g, 99.98%) >Cu^2+^ (2.78 mg/g, 99.06%) >Zn^2+^ (2.19 mg/g, 95.41%) > Ni^2+^ (1.02 mg/g, 49.51%) in mixed-metal solutions as given in S1 Table. At higher pH values, biochar gains more negative charges resulting in metal ion removal through electrostatic attraction. In addition, at higher pH values there is less competition between H^+^ and metal ions for the active sites [39]. The adsorption capacities and removal efficiencies for mixed-metal ions were higher than single-metal ions due to the synergistic effect of mixed-metal ions excluding Ni^2+^. Villaescusa et al. [40] reported that Ni^2+^ had a favorable effect on the adsorption of Cu^2+^ onto grape stalks biochar. Escudero et al. [41] showed that Cu^2+^ had an inhibitory effect on the adsorption of Ni^2+^ when all available active sites were nearly occupied, and Ni^2+^ ions were displaced by Cu^2+^ ions from the adsorbent. Since high adsorption capacities and removal efficiencies were observed for both single and mixed-metal solutions at pH 7, which is also within the range of the pH of actual wastewater, it was selected as the optimum pH for the subsequent experiments. Similar trends were also observed in other studies. Kilic et al. [21] demonstrated that the adsorption capacity of biochar based on almond shell increased from 0 to 10 mg/g for Ni^2+^ and from 10 to 30 mg/g for Co^2+^ when the pH was increased from 2 to 6. Amin et al. [14] found that the removal efficiency of Pb^2+^ by biochar obtained from banana increased from 30% to >90% as the pH was raised from 6–9. The adsorption capacities and removal rates observed from time study and dosage study were given in S1 Table.

### Adsorption isotherms and kinetics

The results from the dosage study were used to test the Freundlich, Langmuir, and Temkin models in linear and non-linear forms. As Table 1 shows, data obtained from the experiments was fitted well by the non-linearized Freundlich isotherm in single-metal solutions (R^2^ = 1, χ^2^ = 0.001–5.6, RMSE = 0.95–3.84) and mixed-metal solutions (R^2^ = 0.99 to 1, χ^2^ =. 0.46–4.20, RMSE = 0.68–3.85) which indicates surface heterogeneity and multilayer adsorption processes [42]. It was observed that the metal ions showed reasonably good adsorption intensities with favorable adsorptions in single-metal solutions (n:1–1.9) and in mixed-metal solutions (n: 2.55–5.44). It can be observed than the adsorption intensities for single-metal ions were lower than those for mixed-metal ions. The closer the n values get to unity, the more heterogeneous the surface is and the more favorable adsorption is. K_f_ values for all the metal ions were in the range of 1.07–6.02 mg/g for single-metal and 0.6–5.95 mg/g for mixed-metal ions. The higher the K_f_ is, the better the adsorption is [35]. It can be noticed that K_f_ values are higher for single-metal ions than mixed-metal ions, indicating that the adsorption is more favorable for single-metal ions than mixed-metal ions as was also the case in another study [35].

**Table 1:** Experimental results along with isotherm model and kinetics parameters for heavy metals adsorption.

		Single-metal,(Mixed-metal)			
		Cu^2+^	Fe^2+^	Ni^2+^	Zn^2+^
Experimental q_e_(mg/g)		2.62,(2.62)	2.58,(2.58)	1.55,(1.4)	2.11,(2.11)
**Isotherm models**					
**Langmuir**	q_m_(mg/g)	5.93,(3.56)	3.94,(3.9)	1.3,(0.73)	3.5,(2.5)
	K_L_(L/mg)	1.62,(12.69)	8,(15)	1.92,(2.49)	1.91,(4.3)
	R^2^	0.99,(0.98)	0.96,(0.94)	0.97,(0.96)	0.99,(0.96)
	χ^2^	1.93,(4.35)	6,(4.11)	0.62,(0.83)	0.1,(0.96)
	RMSE	2.38,(3.08)	3.46,(3.72)	0.98,(0.7)	1.1,(1.43)
**Freundlich**	K_f_ (mg/g)	5.08,(4.97)	6.02,(5.95)	1.07,(0.6)	2.34,(2.33)
	n	1.11,(2.56)	1.9,(2.82)	1,(5.44)	1.28,(2.55)
	R^2^	1,(1)	1,(0.99)	1,(0.99)	1,(0.99)
	χ^2^	1.54,(4.2)	5.6,(3.02)	0.44,(0.55)	0.001,(0.46)
	RMSE	2.36,(3.26)	3.84,(3.85)	0.95,(0.68)	0.99,(1.27)
**Temkin**	B(J/mol)	1.21,(17.31)	3.2,(0.19)	0.07,(0.51)	1.02,(0.9)
	A(L/mg)	17.3,(2.43)	12,(4.86E+06)	9.19E+06,(1.48)	9.91,(13)
	R^2^	1,(1)	0.99,(0.97)	1,(0.99)	1,(0.97)
	χ^2^	2.06,(4.69)	7.92,(4.14)	0.47,(0.57)	0.05,(0.48)
	RMSE	2.39,(4.46)	5.77,(2.98)	0.95,(0.7)	1.08,(1.34)
**Kinetic models**					
**First order**	q_e_(mg/g)	3.24,(3.24)	3.24,(3.24)	1.26,(1.26)	1.26,(1.75)
	k_d_(1/min)	1.73E-02,(1.73E-02)	1.72E-02,(1.73E-03)	0.13,(0.13)	0.13,(0.13)
	R^2^	0.99,(0.99)	0.98,(0.98)	0.99,(0.99)	0.93,(0.99)
	χ^2^	0.01,(0.07)	0.39,(0.39)	2.38E-03,(2.53E-02)	1.32,(5.38E-03)
	RMSE	2.06,(2.24)	2.82,(2.82)	0.67,(0.51)	1.31,(0.99)
**Second order**	k_2_(g/mg/min)	0.557,(1.02)	4.96,(4.96)	0.292,(0.515)	0.0569,(0.382)
	h(mg/g/min)	3.24,(5.88)	31.25,(31.25)	0.47,(0.7)	0.18,(1.09)
	q_e_(mg/g)	2.41,(2.4)	2.51,(2.51)	1.27,(1.17)	1.76,(1.69)
	R^2^	1,(0.99)	1,(0.99)	1,(0.99)	1,(0.99)
	χ^2^	1.03E-04,(1.91E-03)	1.39E-04,(8.01E-05)	1.9E-02,(5.68E-03)	2.58E-02,(4.39E-05)
	RMSE	0.68,(0.66)	0.19,(0.21)	0.6,(0.4)	0.98,(0.91)
**Elovich**	β(g/mg)	7.28,(8.68)	6.28,(6.28)	10.5,(10.5)	5.13,(8.58)
	α(mg/g/min)	2700,(7.53E+04)	2700,(2700)	132,(132)	8,(48.7)
	R^2^	0.98,(0.99)	0.99,(0.99)	0.99,(0.98)	0.97,(0.96)
	χ^2^	0.39,(0.352)	0.18,(0.17)	0.05,(0.1)	0.32,(0.79)
	RMSE	0.77,(0.7)	0.84,(0.83)	0.6,(0.49)	0.76,(0.98)

Kinetics of the heavy metal ion adsorption onto biochar was studied with the pseudo-first, pseudo-second order and Elovich models using effect of contact time data. On the basis of R^2^ (0.99–1), χ^2^ (4.39E-05 to 2.58E-02) and RMSE (0.21–0.98), data set from experiments fitted well to the pseudo-second order model in single- and mixed-metal solutions as shown in Table 1. This indicates that the adsorption of the metal ions onto the biochar is due to chemisorption which involves the exchange, transfer, and sharing of electrons between metal ions and biochar [43]. It was observed that the overall rate constant values (k_2_) < initial rate constant values (h) for metal ions in both single- and mixed-metal solutions, indicating that metal adsorption rate was slow in the beginning and speeded up as time passed [44]. The kinetics data also fits well to the Elovich model with R^2^ ≥ 0.96 in both single- and mixed-metal solutions indicating that the adsorption of the metals onto LF500 biochar is a heterogeneous diffusion process that is controlled primarily by reaction rate and diffusion factor. The α values being higher than the β values indicate that adsorption process is governed by chemisorption [45].

#### Application of pristine biochar blending in aeration tank

Optimum conditions derived from the batch study (0.1 g of biochar, pH 7, contact time of 20 h) were used to remove metal ions from synthetic primary treated wastewater in simulated aeration tank/batch reactor. The highest adsorption capacities and removal efficiencies of the metal ions were Zn^2+^(2.91 mg/g, 93.07%) >Fe^2+^ (0.60 mg/g, 90.04%) >Cu^2+^(0.56 mg/g, 56.15%)>Ni^2+^(0.13 mg/g, 47.83%). These values are lower than the batch study results (1.69–2.92 mg/g, 75–99%). This can be explained by the complexity of the synthetic wastewater with the presence of organics, cations, and anions which compete for the active sites on the limited biochar surface [46, 47]. Nevertheless, with the exception of Ni^2+^, the concentrations of Cu^2+^, Fe^2+^ and Zn^2+^ were well below the WHO drinking water permissible limits (2, 0.3 and 3 mg/L for Cu^2+^, Fe^2+^ and Zn^2+^, respectively) [48]. The removal and adsorption capacity of Ni^2+^ were less than Cu^2+^, Fe^2+^ and Zn^2+^ due to its higher hydrated radius (Ni^2+^:0.302 nm, Cu^2+^: 0.297 nm, Fe^2+^: 0.291 nm and Zn^2+^: 0.295 nm) [49]. Ions with smaller hydrated ionic radius can diffuse onto the adsorbent surface and can accumulate in cracks/channels easily when compared the ions with larger hydration diameters [49]. The results showed that blending biochar in aeration tank reduced metal concentration.

### Fixed-bed column studies

#### Influence of biochar bed height, initial concentrations and flow rate in column study

Higher bed height (1 cm) resulted in extended exhaustion times for both single and mixed-metal solutions because the metal ions had longer time to get into contact with the adsorbent as shown in Table 2 and S1 Fig [50]. Another study also found that *Tectona grandis* leaves biochar exhibited the highest exhaustion time on the removal of Ni^2+^ and Co^2+^ at highest bed depth [27]. Complete breakthrough for Zn^2+^ and Ni^2+^ occurred faster than the other metal ions indicating that biochar has lower adsorption capacity for Zn^2+^ and Ni^2+^. Similarly, it was found that Zn^2+^ and Ni^2+^ were the fastest to breakthrough by alkali-modified biochar derived from hickory wood in a column study [51]. Since Cu^2+^ has a higher hydrolysis constant than Ni^2+^ and Zn^2+^, it has lower degree of solvation allowing it to approach the adsorbent easily (hydrolysis constants for Ni^2+^: 9.86, Zn^2+^: 8.96, Cu^2+^:7.96 Fe^2+^:5.67) [49, 52]. The highest average metal ion uptakes and removal rates for the column with 1 cm bed depth were 5.30 mg/g (39.06%) for Cu^2+^, 8.91 mg/g (43.69%) for Fe^2+^, 4.58 mg/g (34.94%) for Zn^2+^, 1.21 mg/g (10.92%) for Ni^2+^ in single-metal solutions and 3.34 mg/g (23.10%) for Cu^2+^, 3.48 mg/g (23.38%) for Fe^2+^, 1.91 mg/g (17.87%) for Zn^2+^, 1.03 mg/g (10.67%) for Ni^2+^ in mixed-metal solution as shown in Table 2. With higher bed depths, the amount of adsorbent increases and hence, providing a greater surface area and more time for adsorption to take place [53].

**Table 2:** Experimental and modelled parameters from column study for single/mixed-metal solutions.

Conditions					Single-metals, (Mixed-metals)			
Bed depth(cm)	Influent concentration(mg/L)	Flow rate(mL/min)			Cu^2+^	Fe^2+^	Ni^2+^	Zn^2+^
1	5	10		t_e_(min)	300,(300)	360,(300)	240,(180)	240,(180)
			Experimental	Removal(%)	39.06,(23.1)	43.69,(23.38)	10.92,(10.67)	34.94,(17.87)
				q_e_(exp.)	5.3,(3.34)	8.91,(3.48)	1.21,(1.03)	4.58,(1.91)
			Thomas model	q_0_	4.05,(1.85)	6.36,(3.2)	2.34,(0.97)	3.1,(1.92)
				k_th_(L/mg/h)	0.26,(0.41)	0.18,(0.51)	0.19,(0.68)	0.49,(0.69)
				R^2^	0.91,(0.93)	0.97,(0.9)	0.98,(0.86)	0.85,(0.91)
0.5	5	10		t_e_ (min)	240,(180)	240,(180)	180,(120)	180,(120)
			Experimental	Removal(%)	25.25,(17.25)	25.67,(18.47)	10.51,(7.8)	12.71,(12.53)
				q_e_ (exp.)	5.16,(2.6)	6.32,(2.86)	1.59,(0.87)	2.07,(1.63)
			Thomas model	q_0_	2.34,(1.68)	3.32,(1.93)	0.99,(0.59)	0.1,(1.54)
				k_th_(L/mg/h)	0.2,(0.65)	0.32,(0.64)	0.46,(0.78)	0.49,(0.94)
				R^2^	0.85,(0.91)	0.73,(0.89)	0.89,(0.96)	0.97,(0.93)
1	5	20		t_e_(min)	180,(240)	180,(180)	180,(120)	180,(120)
			Experimental	Removal(%)	22.09,(13.32)	23.95,(9.05)	7.96,(6.9)	8.62,(7.38)
				q_e_(exp.)	3.16,(2.57)	2.83,(1.56)	1.14,(0.75)	1.29,(0.76)
			Thomas model	q_0_	5.47,(0.91)	8.06,(2.35)	4.36,(0.92)	3.05,(1.26)
				k_th_(L/mg/h)	0.47,(0.18)	0.38,(0.37)	0.35,(0.45)	0.24,(0.48)
				R^2^	0.96,(0.96)	0.94,(0.94)	0.94,(0.96)	0.98,(0.99)
1	2.5	10		t_e_(min)	540,(480)	540,(480)	420,(360)	480,(420)
			Experimental	Removal(%)	52.14,(35.6)	56.9,(50)	35.5,(12.05)	45.87,(26.76)
				q_e_(exp.)	9.21,(4.25)	9.82,(5.67)	4.82,(1.09)	6.66,(2.78)
			Thomas model	q_0_	6.18,(2.84)	6.73,(4.06)	3.4,(1.34)	3.77,(4.16)
				k_th_(L/mg/h)	0.6,(0.41)	0.38,(0.67)	0.62,(0.72)	0.59,(0.35)
				R^2^	0.93,(0.82)	0.98,(0.93)	0.97,(0.88)	0.86,(0.97)

(t_e_: exhaustion time)

As influent concentration increased to 5 mg/L, breakthrough time was reduced in single and mixed-metal solutions as the metal ions occupy the binding sites faster on the biochar which are easily accessible as shown Table 2 and S1 Fig. The breakthrough curves extended with the increased mass transfer rates which lead to higher removal efficiencies for the lower influent concentrations [54]. For 2.5 mg/L initial metal ion concentration, the highest average metal ion uptakes and removal rates were observed as: 9.21 mg/g (52.14%) for Cu^2+^, 9.82 mg/g (56.90%) for Fe^2+^, 6.66 mg/g (45.87%) for Zn^2+^, 4.82 mg/g (35.50%) for Ni^2+^ in single-metal solutions; 4.25 mg/g (35.60%) for Cu^2+^, 5.67 mg/g (50.00%) for Fe^2+^, 2.78 mg/g (26.76%) for Zn^2+,^ 1.09 mg/g (12.05%) for Ni^2+^ in mixed-metal solutions as shown in Table 2. The removal efficiencies were reduced for mixed-metal solutions as they compete for the available binding sites [5, 53, 55].

Steeper breakthrough curves and decreased exhaustion times were observed at higher flow rate (20 mL/min) for both single- and mixed-metal solutions due to internal mass transfer which increases the diffusion of metal ions [54, 56]. The highest average metal ion uptakes and removal rates for the lower flow rate (10 mL/min) were 5.30 mg/g (39.06%) for Cu^2+^, 8.91 mg/g (43.69%) for Fe^2+^, 4.58 mg/g (34.94%) for Zn^2+^, 1.21 mg/g (10.92%) for Ni^2+^ in single-metal solutions; 3.34 mg/g (23.10%) for Cu^2+^, 3.48 mg/g (23.38%) for Fe^2+^, 1.91 mg/g (17.87%) for Zn^2+,^ 1.03 mg/g (10.67%) for Ni^2+^ in mixed-metal solutions as shown in Table 2 and S1 Fig.

LF500 biochar exhibited less dynamic adsorption capacities and removal rates for mixed-metal solutions because of the competition between metal ions for available binding sites on biochar. In addition, removal rates for the column study were less than the batch study due to limited time for metal ions to interact with biochar surface [57].

#### Adsorption dynamics

The breakthrough curves obtained from the column studies were used to calculate the experimental dynamic adsorption capacities for metal ions. The R^2^ values between the modelled and experimental adsorption capacities for Thomas model ranged from 0.73–0.99 for single-metal and 0.82–0.97 for mixed-metal solutions as shown in Table 2. As the influent concentration was increased for single and mixed-metal solutions, k_th_ values increased [5]. This suggests that less solution is treated with the higher influent concentration resulting in steeper breakthrough curves. There was no correlation between k_th_ or q_0_ with different bed heights. It was also reported that increases in the influent concentrations resulted in lower q_0_ values for single- and mixed-ions metal solutions [5, 58]. When the influent concentration increases, the driving force increases and binding sites are saturated faster, resulting in shortening of the mass transfer zone in which the adsorption occurs [59].

#### Application of pristine biochar in fixed-bed column for secondary treated wastewater

**Fig 1:**
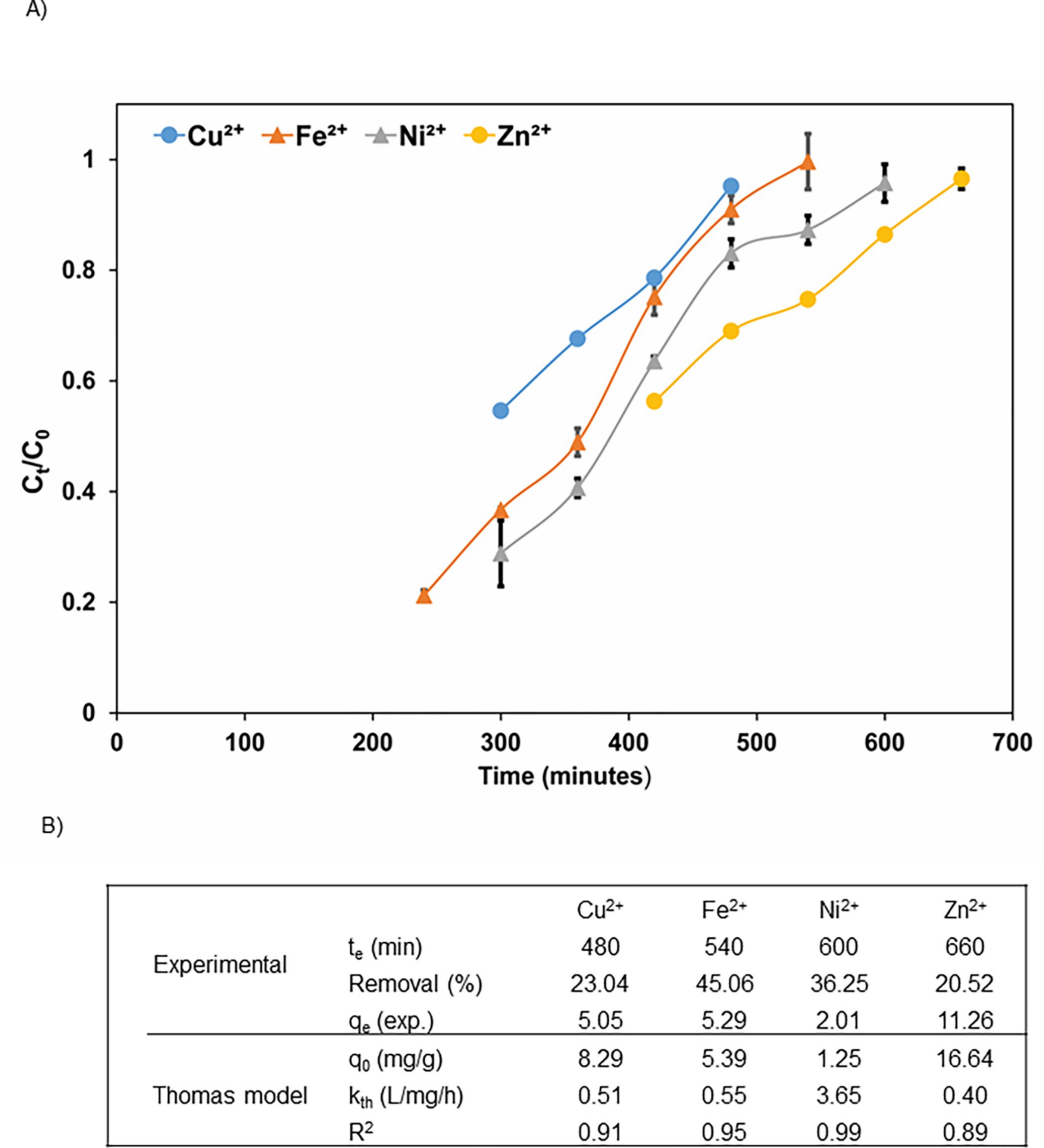
A) Breakthrough curves and B) dynamic adsorption capacities of metals in fixed-bed column in synthetic wastewater (Experimental conditions: pH– 7, bed depth–1 cm, effluent flow rate– 10 mL/min, T– 298 K).

The optimum conditions (1 cm biochar bed depth, 10 mL/min flow rate, pH 7) observed from the fixed-bed column studies were employed to remove metal ions from synthetic secondary treated wastewater in fixed-bed columns. As can be seen in Fig 1, with the exception of Ni^2+^, the adsorption capacities of the metals were increased and the removal efficiencies decreased; 5.05 mg/g (23.04%), 5.29 mg/g (45.06%) and 11.26 mg/g (20.52%) for Cu^2+^, Fe^2+^ and Zn^2+^, respectively, as compared to the fixed-bed studies with single and mixed-metal solutions. This can be explained by synergistic effects of other ions for metal adsorption and different concentrations of the targeted metals (3 mg/L of Cu^2+^, 1 mg/L of Fe^2+^, 4 mg/L of Zn^2+^ and 0.2 mg/L of Ni^2+)^ in wastewater. Zn^2+^ was adsorbed more than Fe^2+^ and Cu^2+^. However, in both single- and mixed-metal solutions Fe^2+^ and Cu^2+^ were the highest adsorbed metal ions. Since Zn^2+^ concentration is less than Cu^2+^ and Fe^2+^, biochar affinity might have increased towards Zn^2+^ due to less competition. The exhaustion times were longer (480, 540 and 600 min for Cu^2+^, Fe^2+^, Ni^2+^ and Zn^2+^, respectively) as compared to the fixed-bed studies with mixed-metal solutions due to competition effect in the presence of other cations and anions. Another study also observed that the exhaustion time increased to 411 min from 396 min in synthetic wastewater for removal of Cr^6+^ from synthetic and real wastewater by a composite of magnetic pine cone [47]. Nevertheless, with this set-up, LF500 biochar was capable of reducing the metal concentrations in secondary wastewater to below the WHO drinking water limits which can be spread into potable aquifers following disinfection.

It was observed that pristine biochar in fixed-bed columns used to remove metals from secondary treated wastewater produced higher adsorption rates than pristine biochar blended in aeration tank to remove metal ions from primary treated wastewater. This can be due to the large concentration gradient consistently present at the interface zone as the solution passes through the column, whereas in the batch reactor there is a decrease in the concentration gradient with time [60].

As can be observed in Fig 1, the Thomas model fit the experimental data with R^2^ = 0.91, 0.95, 0.99 and 0.89 for Cu^2+^, Fe^2+^, Ni^2+^ and Zn^2+^, respectively. Additionally, the adsorption capacities from the experiment were close to the adsorption capacities from the Thomas model, confirming the suitability of the model for the determination of the column dynamics.

#### Regeneration of biochar

**Fig 2:**
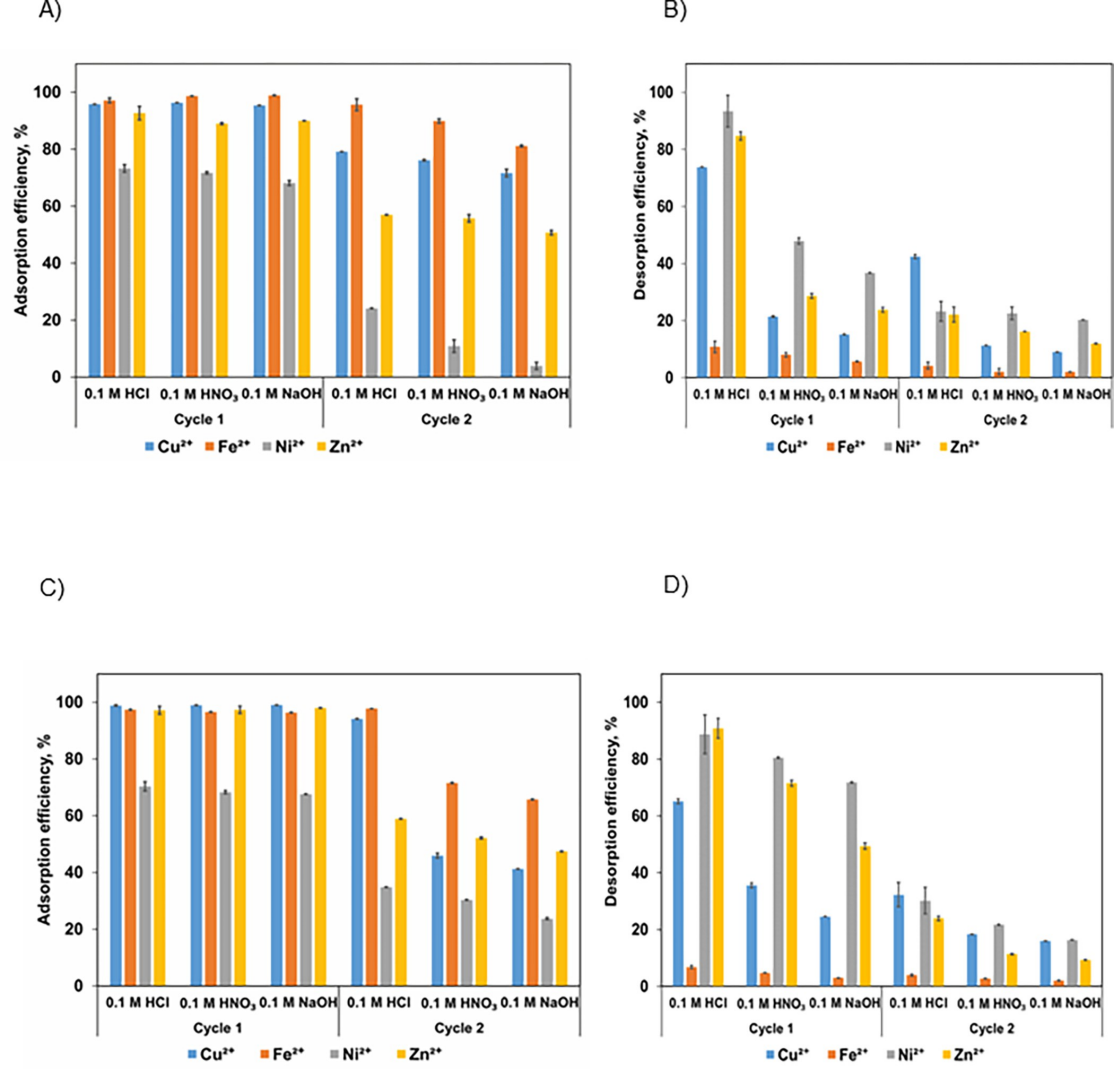
Adsorption efficiency of metals in A) single-metal solution C) mixed-metal solution and their desorption efficiency in B) single-metal solution D) mixed-metal solution (Experimental conditions: pH– 7, Volume– 50 mL, biochar dosage–0.1 g, agitation time– 20 h, agitation speed– 200 rpm, T– 298 K).

Two cycles of adsorption and desorption of the metal ions in single and mixed-metal solutions were conducted to assess the regeneration capacity of the biochar as shown in Fig 2 using different eluents- 0.1 M HCl, HNO_3_ and NaOH. Higher concentrations of eluents were not used due to the possibility that they might damage the structure of LF500 biochar which would in turn decrease the adsorption efficiency [57]. It can be noticed from Fig 2 that 0.1 M HCl was the best eluent for both single and mixed-metal solutions with desorption efficiencies in the first cycle ranging from 10–93% as compared to HNO_3_ (8–80.48%) and NaOH (5.68–71.79%). Chloride ion in HCl has a smaller ionic radius than nitrate in HNO_3_ and hence, HCl was able to elute more metal ions as was also the case in another study [61]. Basic media such as NaOH showed the least desorption potential; bound metal ions were not easy to desorb from the surface of LF500 biochar due to the deprotonation of the coordinating ligands [62]. Hence, 0.1 M HCl was selected as it had the highest desorption potential and did not cause damage to the surface of LF500 biochar.

The desorption efficiencies of Ni^2+^ and Zn^2+^ were highest in both single- and mixed-metal solutions for both cycles as shown in Fig 2B and Fig 2D. The lower desorption capacities for Fe^2+^ and Cu^2+^ can be explained by their high adsorption efficiencies and biochar’s higher affinity for those ions. As a result, it is more difficult to displace them from the adsorption sites. Another study found that the desorption efficiency of Fe^2+^ ions was the lowest (56.37%) as compared to Cu^2+^ (84.51%), Zn^2+^ (79.05%), and Mn (88.65%) due to the natural zeolite’s greater affinity for Fe^2+^ ions [63]. Higher desorption efficiencies of Zn^2+^ and Ni^2+^ can be explained by biochar’s lower affinity for Ni^2+^ and Zn^2+^ which was demonstrated in the earlier equilibrium studies. Therefore, they desorbed easily from biochar. Generally, desorption is much slower than adsorption, and it is likely that a major portion of the adsorbed ions cannot desorb [64]. With time, the retention of adsorbed ions increases due to the formation of stronger bonds, precipitation, and surface/micropore diffusion [35]. Hence, the desorption efficiencies of the second cycle for the metal ions (3–42%) were much lower than the desorption efficiencies of the first cycle (10–93%). Similarly, in mixed-metal solutions, the desorption efficiencies of the second cycle (3–30%) were much lower than the desorption efficiencies of the first cycle (5–90%).

### Characterization of LF500 biochar via SEM, EDX, FT-IR, Boehm’s titration, XPS and XRD

**Fig 3:**
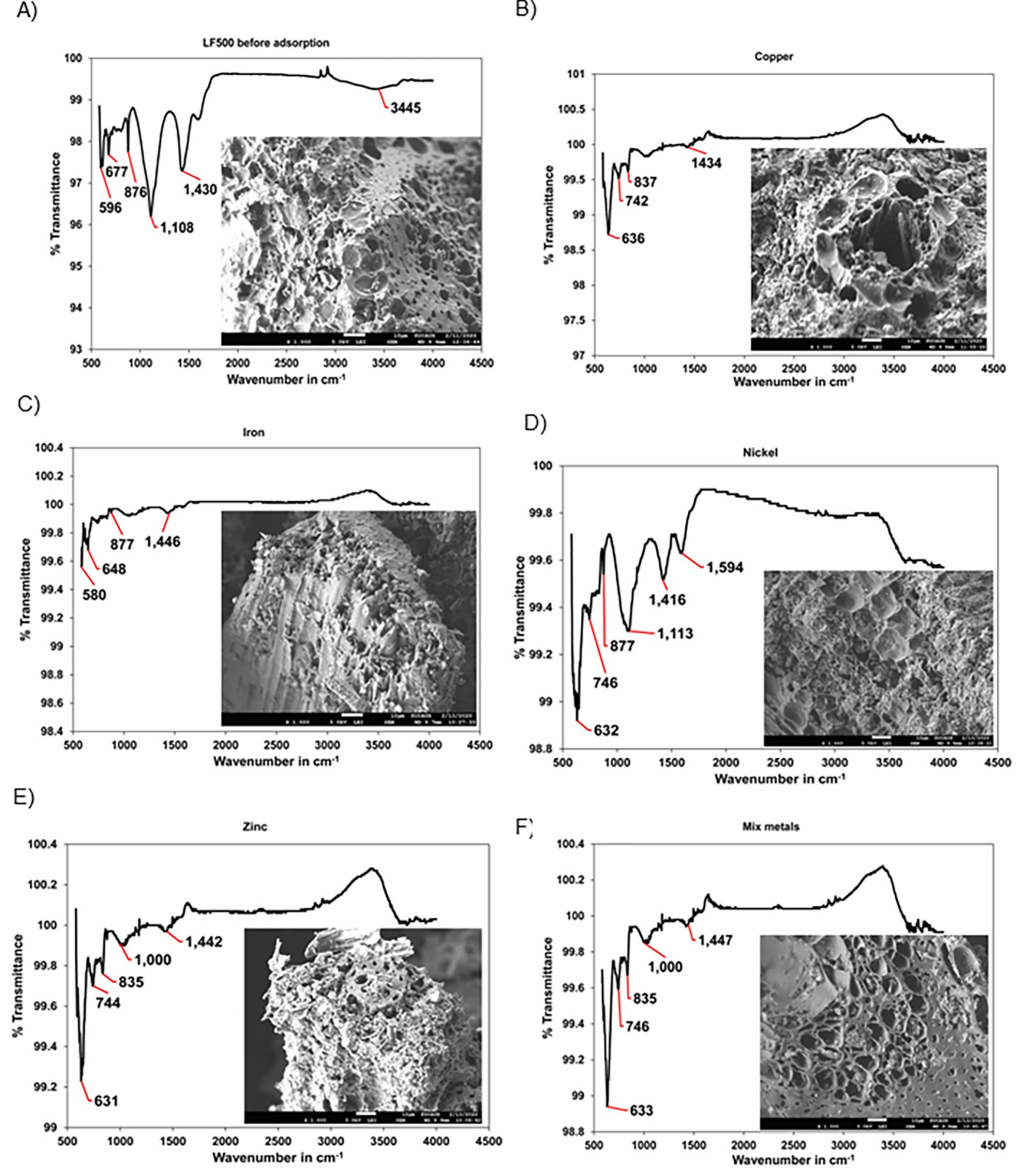
SEM images and FT-IR spectra of date palm LF500 mixture biochar A) before adsorption and after adsorption with B) Cu^2+^ C) Fe^2+^ D) Ni^2+^ E) Zn^2+^ F) mixed-metals.

SEM analysis showed that the LF500 biochar showed porous surface due to matter volatizing during the pyrolysis process before the adsorption, as shown in Fig 3A. However, after adsorption the pores are occupied by the metal ions Cu^2+^, Fe^2+^, Ni^2+^, and Zn^2+^ in single and mixed-metal solutions as shown in Fig 3B–3F, which was also confirmed by EDX analysis. It was found that before adsorption, the biochar did not have any Cu, Fe, Ni, and Zn content. However, after adsorption, the weight (%) of Cu, Fe, Ni and Zn increased to 0.93%, 0.09%, 0.22 and 0.35%, respectively, in single-metal solutions and to 0.34, 0.01, 0.08 and 0.37%, respectively, in mixed-metal solutions.

FT-IR spectroscopy analysis showed the shifts in the oscillation frequencies of carboxyl and aromatic functional groups on the LF500 biochar which were associated with the adsorption of metal ions as shown in Fig 3A–3F. Similar trends were observed by other studies as well [57, 65]. The bands present at 596 and 676 cm^-1^ before adsorption represented alkyne C-H bending vibrations. After adsorption, these bands shifted to 636 and 740 cm^-1^ for Cu^2+^, 580 and 647 cm^-1^ for Fe^2+^, 631 and 745 cm^-1^ for Ni^2+^, 630 and 743 cm^-1^ for Zn^2+^ in single-metal solution, and 632 and 745 cm^-1^ for mixed-metal solution. The band present at 875 cm^-1^ before adsorption represented O-H bending vibrations. After adsorption, the band shifts to 834 cm^-1^ for Cu^2+^, 877 cm^-1^ for Fe^2+^, 876 cm^-1^ for Ni^2+^, 835 cm^-1^ for Zn^2+^ for single-metal solution and 836 cm^-1^ for mixed-metal solution. The band present at 1108 cm^-1^ before adsorption represented C-O stretching vibrations. After adsorption, this band disappears for Cu^2+^ and Fe^2+^, and it is shifted to 1000 cm^-1^ for Zn^2+^ for single and mixed-metal solutions. It is shifted to 1112 cm^-1^ for Ni^2+^ in single-metal solution. Therefore, it can be stated that O-H groups take part in the adsorption of Cu^2+^ and Fe^2+^ [57]. The band present at 1429 cm^-1^ before adsorption represents carbonate groups. After adsorption, this band shifts to 1434, 1445, 1416, 1442, and 1446 cm^-1^ for Cu^2+^, Fe^2+^, Ni^2+^, and Zn^2+^in single-metal and mixed-metal solutions, respectively. A new peak at 1596 cm^-1^ that emerged for Ni^2+^ in single-metal solution was assigned C = C and represented aromatic functional groups.

Results of the Boehm titration indicated that most of the acidic functional groups present on LF500 biochar were carboxylic (1.78 mmol/g) followed by phenolic (1.6 mmol/g) and then lactonic (0.33 mmol/g). This was also in line with another study that used of Douglas fir wood, Douglas fir bark, and hybrid poplar wood-derived biochar [66]. Acidic functional groups such as carboxyl, phenolic as well as hydroxyl functional groups are responsible for the adsorption of metal contaminants [20].

**Fig 4:**
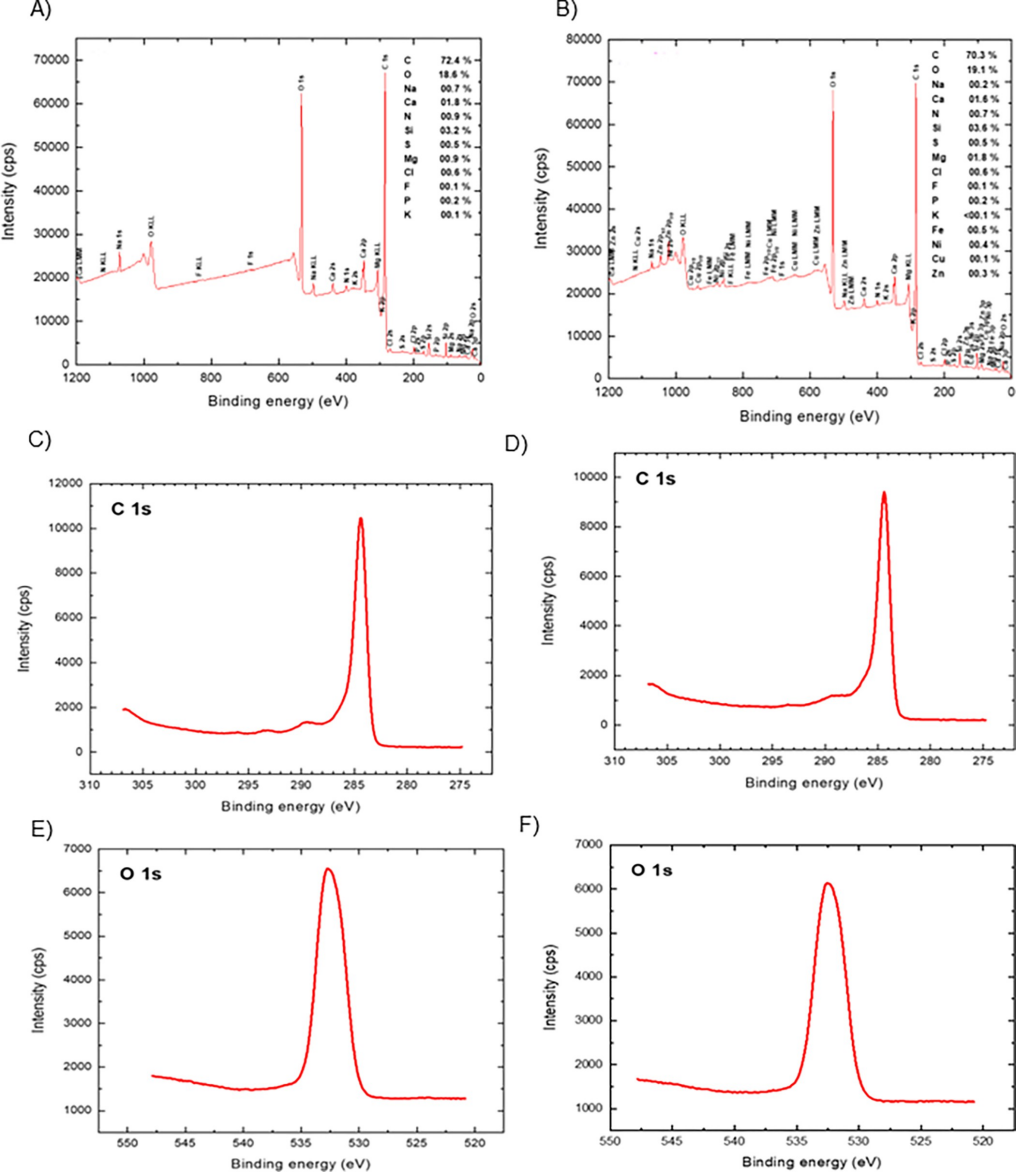
Survey scan XPS analysis of LF500 biochar A) before B) after metals adsorption; high resolution C 1 s spectra C) before D) after metals adsorption; high resolution O 1s spectra E) before F) after metals adsorption.

XPS was used to identify the changes in elemental composition of LF500 biochar as well as changes in its functional group(s) before and after adsorption with metals as Fig 4 shows. According to the XPS survey scan spectra, LF500 biochar was composed of mainly C (72.4%) and O (18.6%) with small amounts of Si (3.2%), Ca (1.8%), Mg (0.9%), N (0.9%) and Na (0.7%). Minimal K, P, F, Cl and S were also detected with amounts under 0.6%. In the survey scan spectra after adsorption, Cu^2+^, Fe^2+^, Ni^2+^ and Zn^2+^ were also detected with amounts of 0.1, 0.5, 0.4 and 0.3% which is similar to the results obtained from the EDX analysis.

From the high-resolution spectra, it can be observed that in the LF500 biochar sample before adsorption, the C 1 s spectrum deconvoluted into one functional group at approximately 285 eV which was assigned to C-H and C-C [67]. Only one peak was also observed in the O 1 s spectra; the binding energy at approximately 532 eV was assigned to organic C-O [67]. After adsorption, the intensity of the peaks in the C 1 s and O 1 s spectra decrease, indicating the possible involvement of the functional groups in the adsorption of the metals. From these results, LF500 biochar is a highly carbonaceous material with an abundance of oxygenated functional groups.

**Fig 5:**
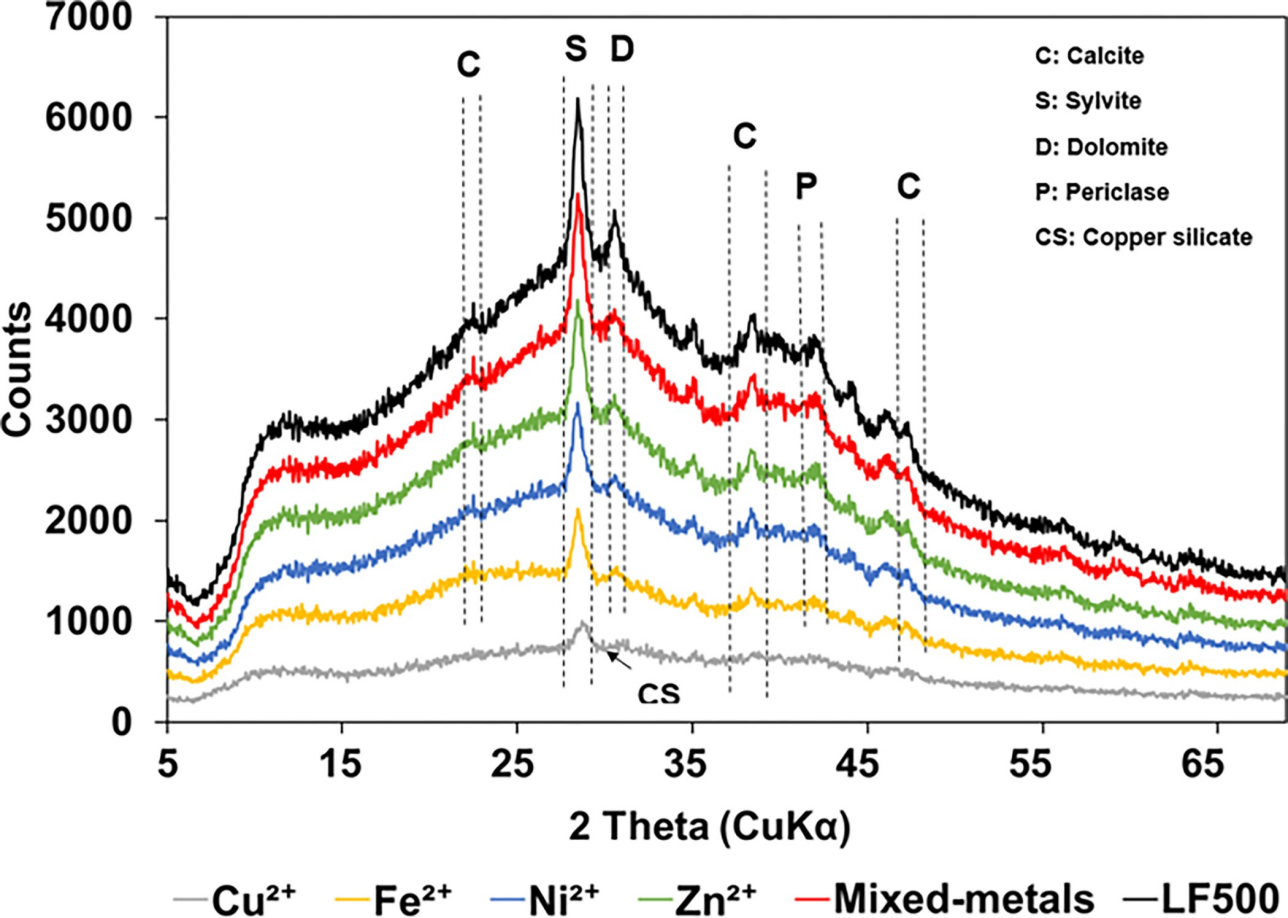
XRD spectra of date palm LF500 biochar before adsorption, and after adsorption with Cu^2+^, Fe^2+^, Ni^2+^, Zn^2+^ and mixed-metals.

The mineral compositions of LF500 biochar before and after adsorption of metals in single- and mixed-metal solutions are depicted by Fig 5. Peaks at 23, 37 and 47° were assigned to calcite [68, 69]. The peaks at 29 and 31° were assigned to sylvite and dolomite, respectively [70]. The peak at 43° was assigned to periclase [71]. The biochar samples had significant amounts of calcite which was observed in the XRD spectra. Calcium was confirmed by the EDX analysis. Carbonate in biochar stems from soil contamination of feedstock during picking and/or the entrapment of CO_2_ during pyrolysis [17]. After the adsorption of the Cu^2+^, some of the peaks that were originally observed in biochar disappeared and a new peak appeared at 30°, indicating that Cu^2+^ precipitated as copper silicate [72]. In contrast, no Ni^2+^ mineral phases were identified for the Ni^2+^ samples and this can be due to the precipitation of Ni^2+^ with amorphous carbon. It is also likely that the XRD diffractometer was unable to pick up the signals from the Ni^2+^ sample owing to its low concentration [69]. After metal adsorption, it was observed that the intensity of the peaks reduces [37, 73].

### Comparison of LF500 with other biochar for the removal of studied heavy metals

LF500 biochar has adsorption capacities comparable to other biochars cited in the literature for the removal of Cu^2+^, Fe^2+^, Ni^2+^ and Zn^2+^ as shown in Table 3. The adsorption capacities tend to vary depending on the source of the biochar and the production conditions [74]. For instance, chicken manure-derived biochar had higher adsorption capacities than olive mill waste for the removal of Cu^2+^ ions as Table 3 shows [75]. Generally, animal waste derived biochars have higher nutrient content, especially potassium and nitrogen, than agricultural waste and hence, tends to adsorb metals more favorably [76]. Moreover, as compared to modified biochar, pristine biochar may have limited adsorption capacities; for instance, 20% H_2_O_2_ activated biochar showed a higher copper adsorption (54 mg/g) than its pristine counterpart (36 mg/g) [77]. However, as compared to pristine biochar, the production of modified biochar can be more expensive, time consuming, complex and involve the use of chemicals; studies have indicated that the production cost can be as high as 37% of that of pristine biochar [78, 79]. LF500 biochar seems to be a promising adsorbent for the removal of Cu^2+^, Fe^2+^, Ni^2+^ and Zn^2+^ according to the results of the batch and column studies performed.

**Table 3:** Adsorption capacities of LF500 and other biochar adsorbents in literature for the removal of studied heavy metals.

Metal	Adsorbent	Adsorption capacity, mg/g		Reference
		Batch study	Column study	
Cu^2+^	Date palm leaf and frond pyrolyzed at 500°C	2.69	9.21	This study
	Corn straw	12.5	NA	[18]
	Hard wood	6.8	NA	
	Dry sewage sludge	6.7	NA	[22]
	Rice husk	0.3	NA	[23]
	Olive pomace	0.7		
	Orange waste	0.4		
	Compost	3.4		
	Hickory wood	2.64	NA	[51]
	Spent coffee grounds	NA	31.15	[26]
	Chicken manure	NA	27.40	[75]
	Olive mill waste	NA	24.87	
Fe^2+^	Date palm leaf and frond pyrolyzed at 500°C	2.92	9.82	This study
	Pristine African beech wood sawdust	2.4	NA	[65]
Ni^2+^	Date palm leaf and frond pyrolyzed at 500°C	1.69	4.82	This study
	Flax shive	19.2	NA	[80]
	Hickory wood	0.24	NA	[51]
	Date seed	5.28	7.33	[81]
	*Tectona grandis* leaves	NA	26.99	[27]
Zn^2+^	Date palm leaf and frond pyrolyzed at 500°C	2.03	6.66	This study
	Corn straw	11	NA	[18]
	Hard wood	4.5	NA	
	Pristine African beech wood sawdust	2.2	NA	[65]
	Water Hyacinth	5.99	NA	[82]
	Spent mushroom compost	6.43–20.41	0.6	[28]

## Conclusion

Due to the simultaneous effect of adsorption and precipitation, the adsorption and removal capacity of LF500 biochar was highest at the pH 7 for Cu^2+^, Fe^2+^, Ni^2+^, and Zn^2+^. Optimum conditions from the batch study (0.1 g of biochar, pH 7, contact time of 20 h) were used to remove metal ions from synthetic primary treated wastewater in simulated aeration tank/batch reactor. Blending biochar in aeration tank reduced metal concentrations in the effluent. The Freundlich model fitted well to experimental results obtained for single and mixed-metal solutions indicating surface heterogeneity and mixed layer adsorption processes. The kinetics study well fitted to the pseudo-second order model and Elovich models in single- and mixed-metal solutions suggesting that the adsorption of the metal ions onto the biochar was due to chemisorption. The presence of metal ions on the biochar after adsorption was confirmed by SEM/EDX studies in addition to changes in the FT-IR, XPS and XRD spectra. Regenerated biochar still had high adsorption and removal efficiency for single and mixed-metal ions. Higher removal efficiencies and dynamic adsorption capacities were observed with lower flow rate, lower influent concentration, and higher bed depth in fixed-bed column studies. The highest average adsorption capacities were observed for 1 cm bed height with 2.5 mg/L of influent concentration and 10 mL/min of flow rate in single and mixed-metal solutions. The optimum conditions observed from the fixed-bed column studies were used to remove metal ions from synthetic secondary treated wastewater in fixed-bed column. LF500 biochar in fixed-bed column was capable of reducing the metal concentrations to below the WHO drinking water limits, which suggests that treated secondary wastewater can be spread into potable aquifers following disinfection. Experimental results fitted well to Thomas models predicting operational parameters in column design for single and mixed-metal solutions and synthetic wastewater. The adsorption capacities and removal efficiencies followed the order of Fe^2+^>Cu^2+^>Zn^2+^>Ni^2+^ in both single- and mixed-metal solutions in both batch and column studies. The order was Zn^2+^ >Fe^2+^>Cu^2+^> Ni^2+^ in synthetic wastewater. It was demonstrated that the pristine biochar is a promising, novel, and cost-effective adsorbent for the removal of metals in wastewater treatment.

## Supporting information

S1 Table Adsorption capacities and removal rates of single and mixed-metal solutions for pH study, dosage study and time study in batch experiments. (PDF)

S1 Fig Effect of bed depth on the removal of Cu^2+^, Fe^2+^, Ni^2+^ and Zn^2+^ in A) single B) mixed-metal ion solutions, effect of influent concentrations in C) single and D) mixed-metal ion solutions and effect of flow rate in E) single- and F) mixed-metal ion solutions (Experimental conditions: pH 7, T– 298 K, biochar bed depth– 0.5 to 1 cm, influent concentration–2.5 to 5 mg/L, effluent flow rate– 10 to 20 mL/min).
(TIF)
