# Supplementary material for: Pristine biochar performance investigation to remove metals in primary and secondary treated municipal wastewater for groundwater recharge application
Source: PLoS One. 2022 Dec 6;17(12):e0278315. doi: 10.1371/journal.pone.0278315 (PMC9725145; doi:10.1371/journal.pone.0278315)
Supplement: S1 Table — (PDF) [file pone.0278315.s001.pdf]

| Study | | Single-metal, Average $q_e$ (mg/g ) and Removal (%) | | | | Mixed-metals, Average $q_e$ (mg/g ) and Removal (%) | | | |
|---|---|---|---|---|---|---|---|---|---|
| | | $Cu^{2+}$ | $Fe^{2+}$ | $Ni^{2+}$ | $Zn^{2+}$ | $Cu^{2+}$ | $Fe^{2+}$ | $Ni^{2+}$ | $Zn^{2+}$ |
| pH | 2 | 0.13,(10.00) | 0.35,(14.20) | 0.21,(10.00) | 0.21,(10.00) | 0.23,(10.00) | 0.13,(10.00) | 0.48,(20.00) | 0.21,(10.00) |
| | 4 | 1.1,(45.11) | 0.73,(29.34) | 0.21,(10.00) | 0.21,(10.00) | 0.23,(10.00) | 0.82,(33.79) | 0.48,(20.00) | 0.21,(10.00) |
| | 6 | 2.24,(91.93) | 2.44,(98.07) | 0.21,(10.00) | 0.21,(10.00) | 2.33,(94.04) | 2.41,(99.17) | 0.41,(21.00) | 0.21,(10.00) |
| | 7 | 2.69,(94.88) | 2.92,(99.15) | 1.69,(75.46) | 2.03,(93.49) | 2.78,(99.06) | 2.94,(99.98) | 1.02,(49.51) | 2.19,(95.41) |
| | 8 | 2.28,(93.43) | 2.46,(98.73) | 1.48,(70.24) | 1.63,(78.09) | 2.44,(98.43) | 2.42,(99.56) | 1.32,(55.27) | 1.85,(89.63) |
| | 10 | 2.28,(93.59) | 2.33,(93.46) | 2.03,(96.00) | 2.04,(97.02) | 2.45,(98.65) | 2.41,(99.03) | 1.32,(55.08) | 1.93,(93.52) |
| Dosage (g) | 0.05 | 5.13,(90.25) | 5.74,(97.45) | 1.90,(42.34) | 3.01,(69.15) | 5.48,(97.42) | 5.77,(97.89) | 1.36,(33.01) | 3.48,(75.87) |
| | 0.1 | 2.69,(94.87) | 2.92,(99.15) | 1.69,(75.46) | 2.03,(93.50) | 2.78,(99.06) | 2.94,(99.98) | 1.02,(49.51) | 2.19,(95.41) |
| | 0.2 | 1.31,(92.43) | 1.39,(94.35) | 0.63,(55.79) | 1.00,(91.61) | 1.34,(95.21) | 1.42,(96.26) | 0.32,(30.83) | 1.07,(93.48) |
| | 0.4 | 0.65,(91.11) | 0.68,(92.55) | 0.34,(60.16) | 0.49,(89.49) | 0.66,(93.52) | 0.70,(94.48) | 0.21,(40.53) | 0.53,(91.94) |
| Time (min) | 1 | 1.63,(79.51) | 1.88,(96.14) | 2.36,(24.47) | 2.38,(30.29) | 0.64,(91.61) | 0.62,(96.96) | 0.82,(23.99) | 1.83,(67.40) |
| | 5 | 1.81,(87.94) | 1.94,(97.06) | 2.38,(28.90) | 2.36,(36.65) | 0.75,(94.36) | 0.69,(96.34) | 1.00,(26.49) | 1.88,(69.06) |
| | 15 | 1.88,(91.47) | 1.97,(97.22) | 2.38,(29.77) | 2.36,(38.40) | 0.77,(95.73) | 0.98,(96.40) | 1.04,(37.57) | 1.98,(73.02) |
| | 30 | 1.93,(93.73) | 1.97,(97.66) | 2.39,(36.90) | 2.38,(40.14) | 0.96,(95.99) | 1.06,(97.24) | 1.09,(40.75) | 2.11,(77.81) |
| | 60 | 1.98,(96.55) | 1.98,(97.74) | 2.39,(59.06) | 2.40,(39.59) | 1.53,(96.35) | 1.15,(97.76) | 1.07,(44.32) | 2.17,(79.74) |
| | 120 | 2.61,(99.43) | 2.62,(99.78) | 2.57,(55.38) | 2.57,(39.97) | 1.27,(99.73) | 1.40,(99.72) | 0.85,(67.79) | 1.56,(73.49) |
| | 240 | 2.62,(99.94) | 2.60,(99.96) | 2.58,(56.55) | 2.58,(70.37) | 1.29,(99.02) | 1.31,(99.86) | 1.50,(58.03) | 2.11,(99.38) |
| | 480 | 2.62,(99.86) | 2.62,(99.95) | 2.58,(55.37) | 2.57,(99.49) | 1.27,(99.96) | 1.43,(99.73) | 2.11,(69.42) | 2.11,(99.46) |
| | 960 | 2.62,(99.93) | 2.62,(99.96) | 2.58,(67.60) | 2.58,(99.48) | 1.55,(99.91) | 1.39,(99.85) | 2.11,(61.82) | 1.95,(91.94) |
| | 1200 | 2.6,(99.08) | 2.62,(98.88) | 2.55,(66.83) | 2.56,(90.75) | 1.53,(99.79) | 1.14,(99.40) | 1.93,(49.51) | 2.09,(98.15) |
| | 1440 | 2.62,(99.64) | 2.62,(99.95) | 2.58,(33.52) | 2.58,(96.97) | 0.77,(99.98) | 1.01,(99.89) | 2.06,(43.52) | 2.00,(94.00) |
| | 2160 | 2.31,(88.02) | 2.62,(99.69) | 2.57,(27.29) | 2.58,(80.24) | 0.63,(99.86) | 0.96,(99.86) | 1.71,(41.07) | 1.70,(80.23) |
