# Supplementary figures and images for: Pristine biochar performance investigation to remove metals in primary and secondary treated municipal wastewater for groundwater recharge application
Source: PLoS One. 2022 Dec 6;17(12):e0278315. doi: 10.1371/journal.pone.0278315 (PMC9725145; doi:10.1371/journal.pone.0278315)

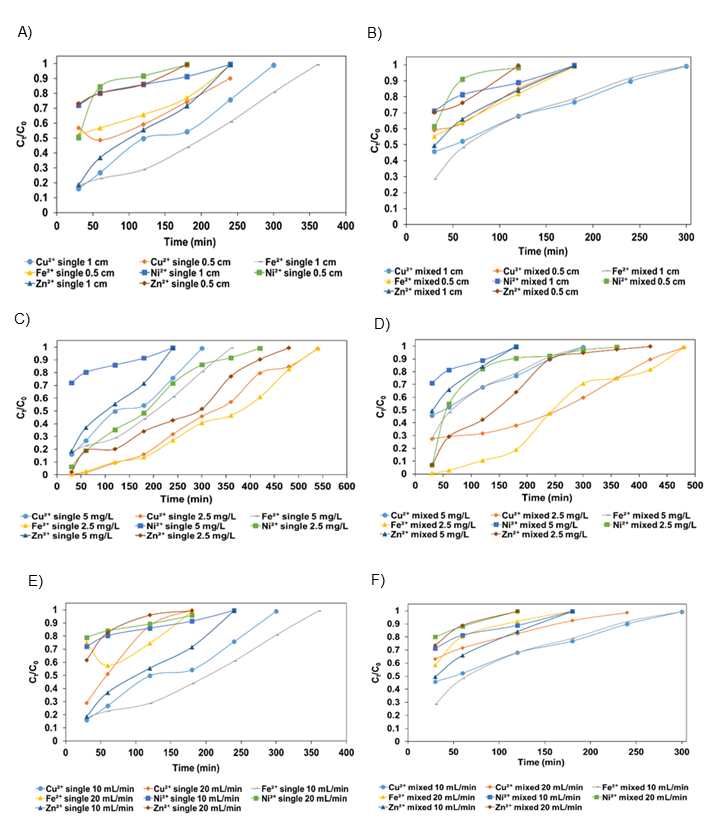

Supplement: S1 Fig — Effect of bed depth on the removal of Cu2+, Fe2+, Ni2+ and Zn2+ in A) single B) mixed-metal ion solutions, effect of influent concentrations in C) single and D) mixed-metal ion solutions and effect of flow rate in E) single- and F) mixed-metal ion solutions (Experimental conditions: pH 7, T– 298 K, biochar bed depth– 0.5 to 1 cm, influent concentration–2.5 to 5 mg/L, effluent flow rate– 10 to 20 mL/min). (TIF) [file pone.0278315.s002.tif]
